# Phytochemical Diversity and Antioxidant Potential of Wild Heather (*Calluna vulgaris* L.) Aboveground Parts

**DOI:** 10.3390/plants11172207

**Published:** 2022-08-25

**Authors:** Vytaute Kaunaite, Gabriele Vilkickyte, Lina Raudone

**Affiliations:** 1 Department of Pharmacognosy, Lithuanian University of Health Sciences, Sukileliu Av. 13, LT-50162 Kaunas, Lithuania; 2 Laboratory of Biopharmaceutical Research, Institute of Pharmaceutical Technologies, Lithuanian University of Health Sciences, Sukileliu Av. 13, LT-50162 Kaunas, Lithuania

**Keywords:** heather, *Calluna vulgaris*, phenolic compounds, antioxidant activity, triterpenic compounds, HPLC

## Abstract

*Calluna vulgaris* L. (heather) is a traditional medicinal plant with anti-inflammatory and calming activities that are determined by the notable amounts of phytochemicals. The evaluation of different populations of plants that accumulate great amounts of bioactive compounds are requisite for the quality determination of plant materials and medicinal and nutritional products. The assessment of natural resources from a phytogeographic point of view is relevant. Phytochemical analysis of heather samples was carried out using spectrophotometric methods and HPLC-PDA techniques, while antioxidant activity was determined using ABTS and FRAP assays. A significant diversification of phenolic and triterpenic compounds and antioxidant activity was determined in the heather samples collected in distinct habitats. Natural habitats, due to their characteristic chemical heterogeneity, lead to the diversity of indicators characterizing the quality of plant raw materials. Chlorogenic acid and hyperoside were found to be predominant among the phenolic compounds, while ursolic, oleanolic acids, and uvaol prevailed among the triterpenic compounds. Thus, these compounds can be suggested as phytochemical markers, characteristic of the heather raw material from central Europe.

## 1. Introduction

*Calluna vulgaris* L. (heather) is an evergreen perennial shrub and the only member of the genus *Calluna* Salisb. belonging to the family Ericaceae Juss [1]. The growth area currently includes northern and western Europe, some parts of Western Siberia, Turkey, Iceland, New Zealand, Morocco, the Azores, North America, Australia, the Mediterranean, and is sometimes found in Asia Minor. This plant grows throughout the territory of Lithuania and occupies more than 10 thousand hectares of area [1,2,3,4,5,6]. The heather is an oligotrophic plant that needs dry, infertile, sandy, acidic (pH = 3.5–6.7), and mineral-poor soil. It grows in pine forests, high bogs, sandy wastelands, burnt and drained peatlands, and near slopes or dunes. Heather is extremely cold-hardy, surviving severe exposure and freezing conditions well below −20 °C [1,2,5,7]. The plant grows in various altitudes and tends to form genetically unique ecotypes [4,8,9].

Heather is used in traditional medicine to treat rheumatism, arthritis, eye diseases, kidney stones, inflammation of the bladder and kidneys, bronchitis, diarrhea, eczema, high blood pressure, increased irritability, anxiety, or sleep disorders. Various pharmacological effects have been reported such as anti-inflammatory, antiseptic, sedative, diuretic, antiviral, cytotoxic, antiproliferative, antibacterial, cardioprotective, hepatoprotective, cytotoxic, and antioxidant effects [3,4,10,11,12,13]. In recent decades, the antioxidant active substances have become the target of research. It is important to identify medicinal plant materials with a high antioxidant potential to produce added-value functional ingredients and to develop herbal medicinal preparations [14,15]. A body of phytochemical compounds, including phenolic and triterpenic compounds, saponins, hydroquinone glycosides, fatty acids, organic acids, amino acids, polysaccharides, sugars, and vitamins have been detected in plant raw materials of *C. vulgaris* [16,17,18,19,20,21]. Phenolic and triterpenic compounds, due to their multi-pharmacological nature, could be responsible for the reported biological effects of the *C. vulgaris*. To the best of our knowledge, the comprehensive analysis of individual specialized metabolites covering phenolic and triterpenic compounds of the same raw materials has not been reported before. Furthermore, the individual populations might possess distinct phytogeographical profiles and markers. Phytogeographical profiling could elucidate the chemical polymorphism and this data could contribute to the knowledge regarding the peculiarities of the phytochemical composition of *C. vulgaris* in natural growing locations. Accumulated bioactive compounds are the main factors determining the quality parameters of raw materials. Only a small part of a medicinal plant species is cultivated and most of the species used in the pharmaceutical industry are collected from natural habitats. Plant heterogeneity is very common in wild populations and has long been used for the selection of medicinal plants [22]. The evaluation of different populations of plants that accumulate large amounts of the active substance is important for selection. Many species of medicinal plants are characterized by interspecific and intraspecific chemical diversity [23]. The study and evaluation of the patterns of diversification are very important for the selection and further cultivation of plants. Plants of the same species may differ not only in quantitative but also in qualitative composition. The increasing demand for plant raw materials increases the exploitation of the natural resources of medicinal plants and leads to the depletion of their resources and the genetic erosion of the species [24]. Resource assessments are very important; therefore, collection activities should be based on assessments of phytochemical parameters of natural habitat raw materials [25]. Cucu et al., 2022, highlight the nectariferous potential of this species, hence the phytochemical profiling could contribute to revealing the pharmacological and nutritional potential of *C. vulgaris* plant materials and honey-related products [26].

Data on the phytochemical composition of heather are lacking both in Lithuania and in other countries of the world. In previous studies, researchers have studied the variation of phenolic compounds in the aboveground part of heather or in the different morphological parts of the plant during flowering [3,4,6,7]. No studies have been performed to evaluate the content or antioxidant activity of heather materials in different natural habitats in temperate climate zone. Furthermore, in the frame of climate change, plants can develop adaptations through the thickening of epicuticular waxes, which can be regarded as a promising source of triterpenes [27]. Therefore, it is important to determine the variability of phenolic and triterpenic compounds and antioxidant activity in heather samples collected in different regions of central Europe.

The aim of this study was to determine the qualitative and quantitative composition of phenolic and triterpenic compounds of the *C. vulgaris*, which grows naturally in the regions of Lithuania, and to evaluate the diversification of antioxidant activity.

## 2. Results

### 2.1. Determination of Total Phenolic and Proanthocyanidin Content

**Figure 1:**
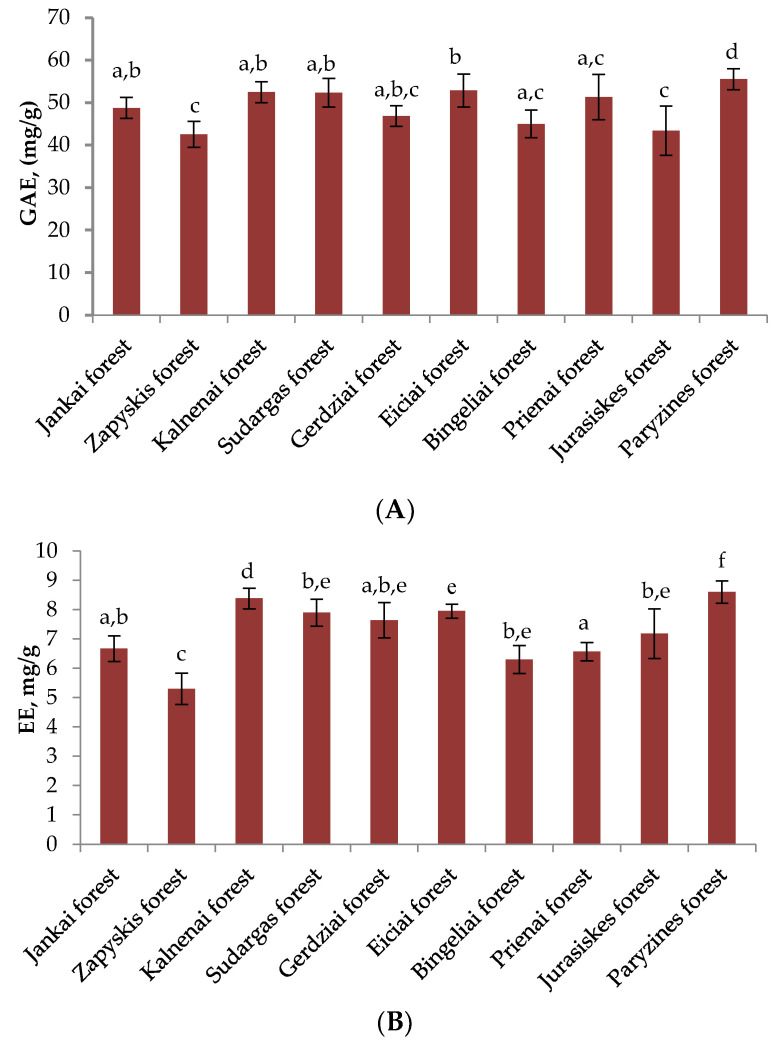
(**A**): variation of total phenolic compounds (mg/g) content of heather samples in different habitats; (**B**): variation of total proanthocyanidins (mg/g) content of heather samples in different habitats. Different letters indicate statistically significant differences between heather samples (*p* < 0.05).

The total content of phenolic compounds in heather samples in different habitats is shown in Figure 1A. The average total content of the phenolic compounds in the collected samples of heather in various habitats was 49.11 ± 5.35 mg/g. The obtained results clearly showed that the highest content of phenolic compounds (55.54 ± 2.48 mg/g) was determined in the plant raw material collected in the forest of Paryzines (Sakiai district). These results significantly differed from the Zapyskis Forest (42.54 ± 3.04 mg/g), the Gerdziai Forest (46.89 ± 2.43 mg/g), the Bingeliai Forest (45.01 ± 3.26 mg/g), and heather samples collected in the Jurasiskes Forest (43.41 ± 5.81 mg/g) (*p* < 0.05). The lowest amounts of phenolic compounds were found in the Zapyskis Forest (42.54 ± 3.04 mg/g) and the Jurašiškes forest (43.41 ± 5.81 mg/g) (*p* < 0.05). The coefficient of variation in the total amounts of phenolic compounds was 5.3%. The results show that the total amounts of phenolic compounds are quite constant between the investigated habitats. 

The variation of the total amount of proanthocyanidins in different habitats is presented in Figure 1B. The average total content of proanthocyanidins in the plant raw material collected in different habitats was 7.25 ± 1.07 mg/g. The highest content of proanthocyanidins (8.6 ± 0.38 mg/g) was identified in the heather samples collected in the Paryzines Forest. These results significantly differed from the plant raw material collected in the Jankai, Zapyskis, Kalnenai, Sudargas, Gerdziai, Bingeliai, and Prienai forests (*p* < 0.05). The samples of heather collected in the Paryzines Forest showed the highest content of proanthocyanidins and phenolic compounds and the strongest radical scavenging activity. The lowest content of both phenolic compounds and proanthocyanidins was determined in samples collected in the Zapyskis Forest (5.3 ± 0.53 mg/g), which significantly differs from the heather collected in the forests of Jankai, Kalnenai, Sudargas, Gerdziai, Eiciai, Bingeliai, Prienai, and Jurasiskes (*p* < 0.05). The content of proanthocyanidins determined in the Zapyskis Forest is 1.6 times lower than the highest content of proanthocyanidins found in the plant raw material collected in the Paryzines Forest. The coefficient of variation was 14.8%.

It is important to evaluate not only the total amounts of compounds but also the amounts of individual specialized metabolites in the different raw material samples. The data can be important in assessing the chemical heterogeneity of the raw material and its possible connections to habitat.

### 2.2. Qualitative and Quantitative Analysis of Phenolic Compounds in Calluna vulgaris Samples

Seventeen individual phenolic compounds belonging to the groups of phenolic acids, flavonols, and flavan-3-ols were identified by the HPLC in all tested plant raw materials collected from different habitats (Table 1). The complex of identified phenolic acids was comprised 4-O-caffeoylquinic acid, 3,5-dicaffeoylquinic acid, chlorogenic acid, neochlorogenic acid, caffeic acid, and protocatechuic acid. Chlorogenic acid was the predominant compound in all samples of different habitats. It constituted about 36–45% of the total amount of phenolic compounds in the above-ground parts of the heather samples. The coefficient of variation of chlorogenic acid in different habitats was 29.35%. The highest content of chlorogenic acid was found in the samples of plant raw material collected in the Paryzines Forest (11,336.9 ± 1524.90 µg/g), meanwhile the lowest in the samples was collected in the Zapyskis Forest (7066.98 ± 1424.99 µg/g). Neochlorogenic acid constituted up to about 9–11% of the total amount of phenolic compounds in the plant raw material. The amounts of neochlorogenic acid were in a range of 1805.94–2620.82 µg/g and no significant differences were determined between the habitats. 4-O-caffeoylquinic and 3,5-dicaffeoylquinic made up about 1–3% of the total amount of phenolic compounds in the plant raw material. The greatest amounts of 4-O-caffeoylquinic and 3,5-dicaffeoylquinic acids were identified in the heather samples collected in the Prienai Forest, respectively, 273.76 ± 17.89 µg/g and 492.2 ± 15.69 µg/g. The content of caffeic acid and protocatechuic acid in the samples of heather was the lowest. Caffeic acid and protocatechuic acid made up only about 1% of the total amount of phenolic compounds in the heather samples.

Six flavonols (astragalin, avicularin, kaempferol, quercetin, isoquercitrin, hyperoside, quercetin-3-arabinopyranoside) and four flavan-3-ols (epicatechin, proanthocyanidins A1, B2, B3) were identified in the heather aboveground samples. The predominant flavonol was hyperoside, which accounted for 11–17% of the total amount of phenolic compounds in the raw material of the heather. The amount of hyperoside determined in the heather samples from the Paryzines Forest was 3951.76 ± 340.93 µg/g, however the significant differences between the habitats were not determined. On average, the amount of hyperoside in the heather samples was—3366.43 µg/g. Avicularin, isoquercitrin, and epicatechin accounted for 3–8% of the total amount of phenolic compounds in the samples of the aboveground parts of the heather. Quercetin, quercetin-3-arabinopyranoside, and astragalin accounted for up to 6% of the total identified phenolic compounds in the heather samples. Of the determined coefficients of variation among the flavonols and catechins, epicatechin (72.97%) varied the most and isoquercitrin (33.82%) and hyperoside (27.19%) the least. The predominant proanthocyanidin in the heather extracts was proanthocyanidin B2, followed by proanthocyanidins A1 and B3. Proanthocyanidin B2 accounted for about 4–6% of the total identified phenolic compounds, meanwhile, proanthocyanidins A1 and B3 up to 1–2%. The highest content of proanthocyanidin B2 was determined in the heather samples collected in the Sudargas Forest (1341.78 ± 124.06 µg/g) and the Bingeliai Forest (1339.4 ± 277.51 µg/g). Among the identified flavan-3-ols, proanthocyanidin B2 varied the least in the plant raw material (35.94%). 

Overall, no differences in phenolic compounds were determined for the samples collected from forest and outskirt habitats, except for the proanthocyanidin B2 and quercetin, where the samples from forest habitats contained significantly greater amounts. The triplet of chlorogenic acid, hyperoside, and neochlorogenic acid was predominant in all test heather samples (Table 1).

### 2.3. Qualitative and Quantitative Analysis of Triterpenic Compounds in Calluna vulgaris Samples

Thirteen triterpenic compounds were detected in the heather samples, namely, oleanolic acid, ursolic acid, maslinic acid, corosolic acid, betulinic acid, betulin, erythrodiol, uvaol, lupeol, β-amyrin, β-sitosterol, α-amyrin, and friedelin (Table 2). The predominant compound in all the samples was ursolic acid. It accounted for 47–55% of the total amount of triterpenic compounds in the plant raw material. The highest content of ursolic acid was found in the samples collected in the Bingeliai Forest (10,709.56 ± 391.94 µg/g) and the Prienai Forest (10,274.12 ± 273.65 µg/g (*p* < 0.05)). The content of uvaol and corosolic acid in the tested samples ranged from 917.87 µg/g to 2450.64 µg/g and from 337.46 µg/g to 793.66 µg/g, respectively. Uvaol accounted for about 6–12% of the total amount of triterpenic compounds in the tested samples, meanwhile corosolic acid constituted up to 4%. The content of α-amyrin in the heather extracts was similar to that of corosolic acid. α-Amyrin was up to 5% of the total amount of identified triterpenic compounds. In the samples of heather, the highest coefficient of variation was found for α-amyrin (55.08%), corosolic acid (54.27%), and uvaol (37.23%) and the lowest for ursolic acid (12.06%). 

Oleanolic acid was the second prevailing triterpenic compound after ursolic acid. The content of oleanolic acid ranged from 3527.23 µg/g to 4795.64 µg/g (coefficient of variation—16.49%), however no significant differences were determined. The amount of β-amyrin was the lowest of all identified oleanane compounds. Erythrodiol, betulin, and maslinic acid accounted for up to 4% of the total amount of triterpenic compounds.

The content of betulinic acid ranged from 404.34 µg/g to 812.32 µg/g in samples collected at different habitats. The results did not differ statistically significantly from the plant raw material collected in other habitats. Amounts of friedelin and lupeol ranged from 85.52 µg/g to 221.33 µg/g and from 0.27 µg/g to 16.90 µg/g, respectively. These compounds made up about 1% of the total identified triterpenic compounds. β-Sitosterol was the only steroid compound detected in the assay; its amount ranged from 251.83 ± 20.67 µg/g to 382.73 ± 8.89 µg/g. 

Great intrapopulational variabilities were determined for all identified triterpenic compounds. Furthermore, no significant differences were determined between the samples of heather collected in the outskirt habitats and the forest habitats. On the other hand, ursolic acid, oleanolic acid, and uvaol prevailed in all samples of the tested populations.

### 2.4. Antioxidant Activity of Heather Herb Extracts

**Figure 2:**
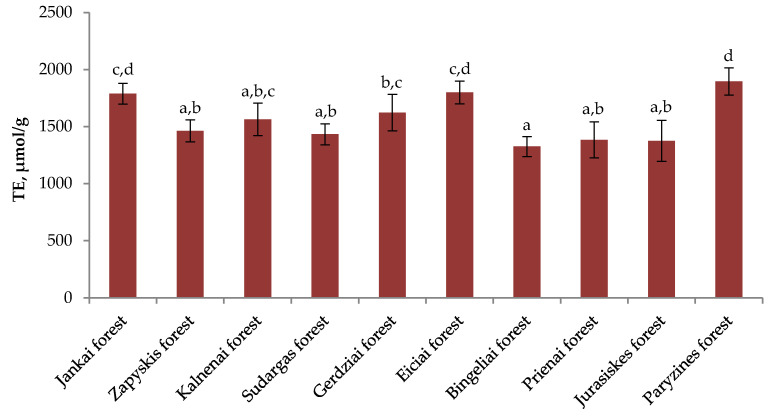
The radical scavenging activity (TE, µmol/g) in different Lithuanian wild habitats; different letters indicate statistically significant differences between heather samples (*p* < 0.05).

The variation of radical scavenging activity in heather samples from different sites is shown in Figure 2. The average radical scavenging activity of different habitats was 1565.48 µmol/g. Samples of heather collected in the forest of Paryzines (1896.4 ± 118.79 µmol/g) presented the highest radical scavenging activity and they differ statistically significantly from the Zapyskis Forest (1463.3 ± 96.97 µmol/g), the Kalnenai Forest (1564 ± 142.83 µmol/g), the Sudargas Forest (1433.2 ± 92.59 µmol/g), the Gerdziai Forest (1623.5 ± 160.59 µmol/g), the Bingeliai Forest (1325.7 ± 87.62 µmol/g), the Jurasiskes Forest (1375.8 ± 179.69 µmol/g), and the Prienai Forest (1384.6 ± 158.32 µmol/g) of collected heather samples (*p* < 0.05). The lowest radical scavenging activity was observed in the samples of plant raw material collected in the Bingeliai Forest (1325.7 ± 87.62 µmol/g). These results differ statistically significantly from the Jankai Forest (1788.8 ± 91.75 µmol/g), the Gerdziai Forest (1623.5 ± 160.59 µmol/g), the Eiciai Forest (1799.5 ± 99.9 µmol/g), and heather samples collected in the Paryzines Forest (1896.4 ± 118.79 µmol/g) (*p* < 0.05). The coefficient of variation of radical scavenging activity of heather aboveground parts growing in different habitats is—14.3%. 

**Figure 3:**
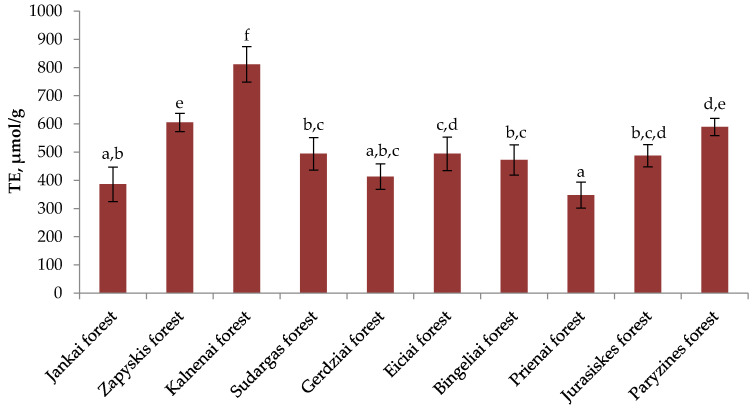
Variation of reducing activity (TE, µmol/g) in different Lithuanian wild habitats; different letters indicate statistically significant differences between heather samples (*p* < 0.05).

The results provided in Figure 3 demonstrate the reducing activity of heather raw material in different natural habitats. The average reducing activity of heather raw material in different habitats was 510.308 µmol/g. The strongest reducing activity was found in the samples collected in the Kalnenai Forest (811.47 ± 63.14 µmol/g), which differed significantly from the samples of plant raw material collected in the Jankai, Zapyskis, Sudargas, Gerdziai, Eiciai, Bingeliai, Prienai, Jurasiskes, and Paryzines forests (*p* < 0.05). Samples of heather collected in the Prienai Forest (347.87 ± 46.05 µmol/g) had the lowest reducing activity, which showed almost two-and-a-half times lower activity than the highest reducing activity found in the Kalnenai Forest. These results differed significantly from the reducing activity of plant raw material extracts collected in the Zapyskis, Kalnenai, Sudargas, Eiciai, Bingeliai, Jurasiskes, and Paryzines forests (*p* < 0.05). The lowest variation of reducing activity was found in the Paryzines Forest collected raw material—5.26%.

The correlation analysis was performed between the radical scavenging activity, the reducing activity, and the determined amounts of phenolic origin compounds. There was a moderate positive correlation between total proanthocyanidins and radical scavenging activity (R = 0.423, *p* < 0.05), but a weak correlation between proanthocyanidins and reducing activity (R = 0.286, *p* < 0.05). The total amount of phenolic compounds in the heather aboveground parts well correlated with the radical scavenging activity (R = 0.521, *p* < 0.05). Furthermore, the antioxidant activities of heather herb extracts were correlated to the amounts of individual phenolic constituents. A significant correlation was found between reducing activity and the contents of protocatechuic acid (R = 0.444, *p* < 0,05), avicularin (R = 0.348, *p* < 0,05), chlorogenic acid (R = 0.427, *p* < 0,05), isoquercitrin (R = 0.286, *p* < 0,05), and kaempferol (R = 0.314, *p* < 0,05) between radical scavenging activity and the contents of 3,5-dicaffeoylquinic acid (R = 0.328, *p* < 0.05), proanthocyanidin B3 (R= 0.431, *p* < 0.05), quercetin (R = 0.392, *p* < 0.05) between hyperoside and radical scavenging activity (R = 0.404, *p* < 0.05). Reducing activity was correlated with chlorogenic acid (R = 0.338, *p* < 0.05). 

### 2.5. Principal Component Analysis

**Figure 4:**
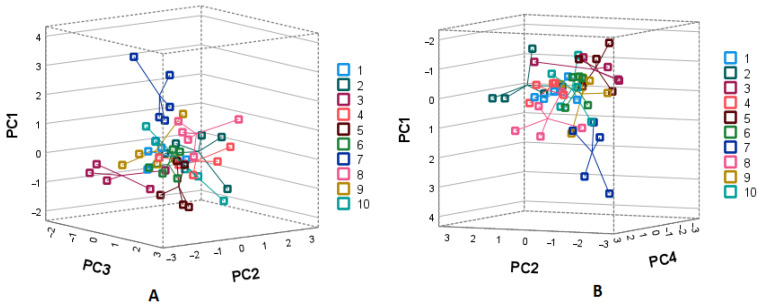
PCA loading plots of triterpenic compound variables ((**A**)—PC1, PC2, and PC3; (**B**)—PC1, PC3, and PC4). 1—Jankai; 2—Zapyskis; 3—Kalnenai; 4—Sudargas; 5—Gerdziai; 6—Eiciai; 7—Bingeliai; 8—Prienai; 9—Jurasiskes; 10—Paryzines.

**Figure 5:**
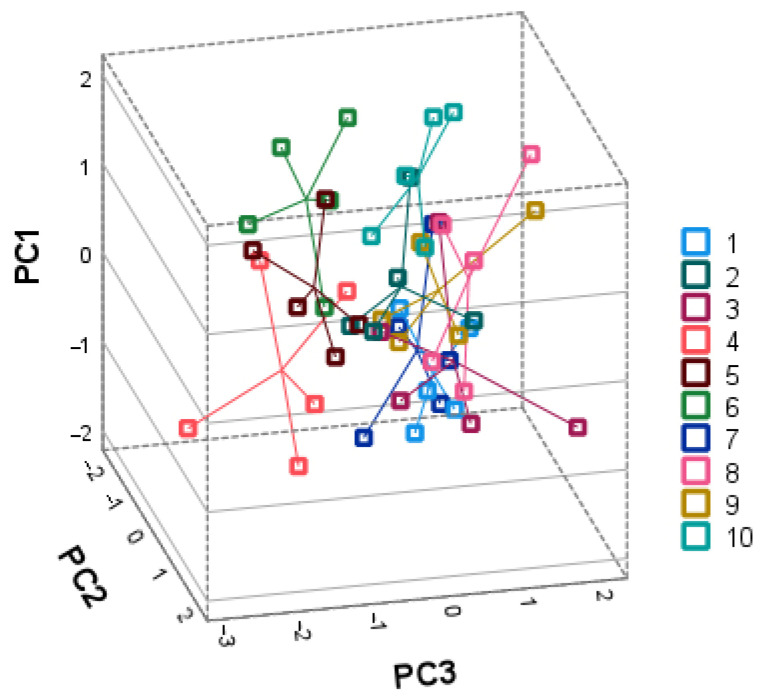
PCA loading plots of phenolic compound variables. 1—Jankai; 2—Zapyskis; 3—Kalnenai; 4—Sudargas; 5—Gerdziai; 6—Eiciai; 7—Bingeliai; 8—Prienai; 9—Jurasiskes; 10—Paryzines.

PCA was used to isolate the major components to investigate the relationships between triterpenic and phenolic compounds to determine the primary predictors between the intrapopulation and interpopulation samples studied. The loads revealed characteristic variables for each group of identified compounds in a graph depicting the data in the 3D scatter graphs composed of the major components (Figure 4 and Figure 5). 

The first PCA model was constructed using identified triterpenic compounds (Figure 4). Four principal components covering 62.42% of the total data variance were extracted. The first principal component (PC1) explained 19.98% of the total variance. PC1 positively correlated with the amounts of maslinic acid (0.757) and corosolic acid (0.847) and negatively with betulin (−0.718). The PC2 positively correlated with uvaol and α-amyrin (0.885 and 0.793, respectively). The PC3 positively correlated with oleanolic acid, erythrodiol, and friedelin (0.691, 0.594, and 0.751, respectively) and negatively with betulinic acid (−0.500). The four principal components well correlated negatively with β-amyrin (−0.893) and positively with β-sitosterol (0.824). Figure 4 presents the PCA1 score plots. The first principal component separates all the accessions from the Bingeliai Forest (population no. 7). The samples contained the greatest amounts of maslinic and corosolic acids and above average amounts of ursolic and oleanolic acids. On the other hand, the samples from Kalnėnai (no. 3) distanced at the lower position to PC1 and distinguished with the lowest amounts of α- and β-amyrins, friedelin and erythrodiol and with a lower fraction of prevailing compounds, ursolic and oleanolic acids. The samples from Zapyskis, Kalnenai, and Gerdziai (populations numbers 2, 3 and 5, respectively) contained the greatest amounts of betulin and high levels of oleanolic acid and were slightly distanced from all other samples. The third principal component on the negative side differentiates the samples from Zapyskis (population no. 2). However, only the samples from Bingeliai showed clear separation from other populations. This habitat was situated in the most southern part of Lithuania. The other samples from different habitats showed high intrapopulational diversity in the amounts of triterpenic compounds. 

The second PCA model was constructed using identified phenolic compounds (Figure 5). Three principal components characterized 61.31% of the total variance. The first principal component explained 28.56% of the total variance and was positively correlated with the amounts of avicularin, hyperoside, isoquercitrin, and quercetin-3-arabinopyranoside (0.832, 0.671, 0.527 and 0885, respectively). The first principal component separates the samples collected in Eiciai (no. 6) and Paryzines (no. 10). The second principal component accounted for 18.42% of the total variance and was positively correlated with astragalin (0.660), epicatechin (0.756), and proanthocyanidin B2 (0.785). The second principal component discriminated the Sudargas samples (no. 4) with notable amounts of epicatechin and proanthocyanidin B2. The third principal component was correlated with chlorogenic (0.579) acid and isoquercitrin (0.552) and negatively with quercetin (−0.752). The third component differentiated the samples into three slightly overlapping groups. The first group coupled population numbers 4, 5 and 6 (Sudargas, Gedziai, and Eiciai, respectively). This group can be characterized by the greatest amounts of quercetin. The second group contained samples from population no. 10 (Paryzines). The third group coupled the populations nos. 1, 2, 3 and 7, 8, 9. All these habitats were located in the western part (Jankai no. 1, Zapyskis no. 2 and Kalnenai no. 3) and the southern part of Lithuania (Bingeliai no. 7, Prienai no. 8, and Jurasiskes no. 9). The samples of these populations were scattered along the PC1 and positive side of PC3 and showed high intrapopulational variability. 

## 3. Discussion

This study revealed the phytochemical composition of *Calluna vulgaris* aboveground part samples in wild populations, as well as significant intrapopulational variability. The phytochemical composition of the heather aboveground parts depends on the growing region. The significant differences were determined between the distanced habitats in the western and southern parts of Lithuania. The quantitative and qualitative compositions of total phenolic compounds, as well as individual phenolic and triterpenic compounds along with the antioxidant activity in different habitats of central Europe, were determined for the first time. Plants with diverse phytochemical compositions have become the target of interest due to the emerging data of their high-antibacterial capacity [28]. Mandim et al., determined that phenolic-rich fractions are the most potent antibacterial agents, with the ability to sustain the commensal flora [16]. Rodrigues et al., compared the total variation of phenolic compounds in the heather raw material using different solvents and determined that the total content of the phenolic compounds in different heather extracts ranged from 53.96 mg/g DW to 121.92 mg/g DW. The highest content of phenolic compounds was found in the hydroalcoholic heather extract (121.92 ± 0.815 mg GAE/g DW) [17]. In another study by Vučić et al., the results of total phenolic content ranged from 67.55 ± 0.38 mg GAE/g of extract to 142.46 ± 0.50 mg GAE/g of extract. The aqueous extract had the highest content of phenolic compounds (142.46 ± 0.50 mg GAE/g of extract), meanwhile ethyl acetate extract had the lowest content of phenolic compounds (67.55 ± 0.38 mg GAE/g of extract) [12]. In our study, the total content of phenolic compounds in hydroalcoholic heather extracts ranged from 42.54 mg/g to 55.54 mg/g in different habitats. Comparing the results of this study with data published by other researchers, some differences were observed, which might be explained by distinct geographic growing conditions as the raw materials in the above-mentioned studies were collected from the southern parts of Europe [12,17]. 

The scientific literature regarding the variability of proanthocyanidins in the heather raw material is still scarce. Proanthocyanidins, like other phenolic compounds, have a very strong antioxidant effect. Phenolic compounds protect the body from the effects of harmful reactive oxygen and nitrogen species, oxidative stress, and the progression of chronic diseases. These compounds reduce low-density lipoprotein cholesterol but increase high-density lipoprotein cholesterol and have antiviral, antibacterial, anti-inflammatory, anti-allergic, and anticarcinogenic effects [29,30]. The content of proanthocyanidins in heather depends on the peculiarities of the habitats and their highest amounts are determined during the flowering period in the flower part [3,4]. In this study, the total amount of proanthocyanidins among different habitats ranged with values from 0.53 mg/g to 8.6 mg/g. Proanthocyanidins significantly impact antioxidant activity [15]. Chepel et al., identified a weak correlation between proanthocyanidin content and reducing activity (R = 0.270). Chepel et al., showed that the total content of proanthocyanidins in the raw material of heather during the flowering period in different parts of the plant varied from 3.98 ± 0.21 to 9.18 ± 0.11 mg/g [3]. Thus, the variability of proanthocyanidins in plant raw material is similar when compared to other researchers’ studies. Heather raw material, as a rich source of proanthocyanidins, could be further selected as a target material for the purification of proanthocyanidins and their biological activity verification. 

It is important to elucidate high antioxidant potential plant materials for the production of the added-value functional ingredients to cope with the aging processes [31]. Rieger et al., using the DPPH assay, determined that heather extracts possessed the greatest radical scavenging activity compared with *Sambucus nigra* flowers and fruits and *Vaccinium myrtillus* fruits [8]. The total amount of phenolic compounds impacts the radical scavenging activity. Varga et al., determined that heather extracts could be perspective antioxidant and antimicrobial agents [32]. Antioxidant activity fluctuates depending on the individual plant ontogenesis. Studies confirm that massive flowering ensures the greatest amounts of antioxidants [3]. Furthermore, massive flowering is the most attractive nectariferous period for pollinators, who ensure the apitherapeutical potential of *C. vulgaris* [33]. Various studies confirm the outstanding antioxidant activity of *C. vulgaris* honey due to its rich and diverse phenolic content [26]. 

Chlorogenic acid was the predominant compound in plant raw material with a relatively low coefficient of variation. In previous studies, chlorogenic acid has been reported to be a major compound in heather extracts as well [6,34]. Thus, chlorogenic acid can be regarded as a phytochemical marker of heather, regardless of the collection site. Lower amounts of other hydroxycinnamic acids were determined, such as 4-O-caffeoylquinic acid, 3,5-dicaffeoylquinic acid, neochlorogenic acid, and caffeic acid. Drozdz et al., detected amounts of caffeic acid ranging from 1.39 ± 0.26 µg/g to 9.60 ± 0.61 µg/g. Meanwhile, in our study, it was higher and ranged from 43.28 ± 3.66 µg/g to 50.42 ± 6.95 µg/g [6]. Among all identified phenolic compounds, the lowest amount was found for protocatechuic acid. On the other hand, other phenolic acids, i.e., ferulic acid and p-coumaric acid, have been identified in various studies; meanwhile, in our study, they have not been identified [6]. Mandim et al., determined that 5-O-caffeoylquinic acid is the only phenolic acid in Portuguese heather samples [28]. In another study, researchers identified 5-O-caffeoylquinic and 5-p-coumaroylquinic phenolic acids [16]. These compounds were not predominant in the Portuguese heather samples. Meanwhile, Vostinaru et al., have found that the main compounds are flavonoids and chlorogenic acid in Romanian heather samples [35]. Scientific data suggests that heather accumulates great amounts of flavonoids, whose profiles seem to have significant phytogeographical characters. The predominant flavonoid in this study was hyperoside. Orhan et al., have reported that the predominant flavonoid in heather extracts collected from northeastern Turkey is kaempferol-3-O-β-D-galactoside [34]. Rodrigues et al., have found that the predominant compounds are myricetin glycoside and quercetin [17]. Myricetin-rhamnoside was a prevailing flavonoid in the Portuguese heather samples [16]. Tarchenko et al., determined that chlorogenic acid predominated among hydroxycinnamic acids; hyperoside, rutin, and quercetin-3-D-glucoside predominated among flavonoids; and among tannin metabolites, (−)-epigallocatechin and (+)-gallocatechin were the prevailing compounds [7]. The low amounts of kaempferol obtained in this study were consistent with previous studies. Rodrigues et al., reported a kaempferol level of 15.36 ± 0.11 µg/g and this corresponds to the lowest amount of kaempferol found in our study [17]. Comparing this study with others, flavonoids such as rutin, luteolin, apigenin, isorhamnetin, quercitrin, etc., were additionally detected [6,16,17]. Monschein et al., 2010 and Rieger et al., 2008 found that the amounts of flavonol glycosides in heather increase with altitudes of habitat [4,8]. The individual compound similarities and differences between wild heather populations occur in different habitats, suggesting the existence of different geo chemotypes. 

The distribution of triterpenic compounds in the heather raw material has been little studied. Yet, the investigation of the rich sources of triterpenic compounds is an important task of applied research. Triterpenoids have antioxidant, cardioprotective, anti-inflammatory, hepatoprotective, antidiabetic, anticancer, and antiviral effects [10,36]. Furthermore, the triterpenic compounds can often be regarded as chemophenetic markers at the level of the genus [37,38,39]. The predominant compounds in the raw material of heather are ursolic acid and oleanolic acid. These acids have similar biological activity and the effects mentioned above. The lower amount of triterpenic compounds besides ursolic and oleanolic acids were identified as uvaol, betulinic acid, α-amyrin, corosolic acid, maslinic acid, betulin, β-sitosterol, erythrodiol, friedelin, β-amyrin, and lupeol, and comprised characteristic quantitative profiles. Szakiel et al., were the first to provide a comprehensive review of the triterpenoid composition of heather cuticular waxes in flowers and leaves. Triterpenic acids were determined to be the predominant compounds, especially in leaf cuticular wax, where they accounted for 83% of total triterpenoids, meanwhile in flowers 56%. The results of a study conducted by Szakiel et al., showed that ursolic acid is the major triterpenic compound in the heather cuticular waxes of flowers and leaves. It constitutes up to 61% of the total amount of triterpenic compounds in the cuticular waxes of leaves and 37% in the flowers [5]. Comparing the data of this study with the data of Szakiel et al., it can be stated that the percentage of ursolic acid is similar in both studies. Furthermore, particular Ericaceae plants could be a promising source for the purification and elucidation of new triterpenic compounds. 

The three-dimensional distribution plot of PCA analysis is a powerful tool, coupling abundant phytochemical data and elucidating distributional patterns between analyzed samples [40]. The PCA plotting revealed particular patterns of distribution of heather populations due to possible differences in location of habitats and distinguished the groups with the particular chemical compositions of interest. Our results revealed great intrapopulational variations and elucidated two habitats with notable amounts of quercetin. The latter compound can be a potent reversible inhibitor of monoaminooxidase-A and might be responsible for the neurostabilizing effect of heather [41].

## 4. Materials and Methods

### 4.1. Chemicals and Solvents

Distilled water was purified using a Milli–Q system (Millipore, Bedford, MA, USA). Ethanol (96%) was obtained from Vilniaus degtine (Vilnius, Lithuania). Folin–Ciocalteu reagent, acetic acid (99.8%), and hydrochloric acid (37%) were purchased from Sigma–Aldrich (Buchs, Switzerland). The following reagents were used: sodium carbonate (Na_2_CO_3_), gallic acid, 2,2′-azino-bis(3-ethylbenzothiazoline-6-sulfonic acid) diammonium salt (ABTS), 6-hydroxy-2,5,7,8-tetramethylchroman-2-carboxylic acid (Trolox), potassium persulfate (K_2_S_2_O_8_), sodium acetate (CH_3_COONa), 2,4,6-Tri-(2-pyridyl)-S-triazine (TPTZ), 4-dimethylaminocinnamaldehyde (DMCA), (−)-epicatechin, and ferric chloride hexahydrate (FeCl_3_ × 6 H_2_O) from Sigma-Aldrich.

Analytical and chromatographic grade solvents and standards were used for this study: acetonitrile, methanol, trifluoracetic acid, α-amyrin, β-amyrin, β-sitosterol, lupeol, erythrodiol, maslinic acid, oleanolic acid, isoquercitrin, proanthocyanidins A1 and B2, epicatechin, caffeic acid, protocatechuic acid, quercetin, quercetin-3-arabinopyranoside, kaempferol, astragalin, avicularin, neochlorogenic (5-O-caffeoylquinic), chlorogenic (3-O-caffeoylquinic), 4-O-caffeoylquinic, and 3,5-dicaffeoylquinic acids from Sigma-Aldrich; uvaol, friedelin, betulin, betulinic acid, corosolic acid, hyperoside, and proanthocyanidin B3 from Extrasynthese (Genay, France); ursolic acid from Carl Roth (Karlsruhe, Germany).

### 4.2. Plant Materials

*Calluna vulgaris* aboveground parts were collected from 10 different Lithuanian habitats in August 2020 during massive flowering. The plant species was identified according to the morphological characteristics by Vytautė Kaunaitė and Lina Raudonė. The voucher specimen was deposited in the herbarium of Vilnius University Siauliai Academy (HUS). Samples of five individual plants were collected at each different habitat (Table 3). The meteorological conditions (temperature (°C), precipitation (mm), and sunshine duration (h)) and soil characteristic data of the growth sites were obtained from the Lithuanian Hydrometeorological Service and the Soil Atlas of Europe [42,43] and are presented in Tables S1–S4. According to the climate classification, Lithuania belongs to the continental climate category Dfb. Temperatures in summer are in the range of 21–32 °C during the day and 10–18 °C at night. Average winter temperatures are in the range of −12–7 °C during the day and −23–−4 °C at night [44]. The raw material was dried at room temperature in a well-ventilated chamber and protected from direct sunlight and moisture. The dried raw material was milled to powder passing through the sieve number 355 and stored in a dark dry place.

### 4.3. Sample Preparation

The heather herb extracts were prepared using 0.20 g of dried raw material and 20 mL of 70% ethanol. The samples were extracted in an ultrasonic bath at 25 °C for 15 min. The extracts were centrifuged for 15 min at 6800 rpm and filtered through a 0.22 μm pore size filter. The samples were stored in a dark place protected from light.

### 4.4. Determination of Total Phenolic Content

The total phenolic content was determined by the Folin–Ciocalteu method [45]. The test solution was prepared by mixing 20 μL of each extract with 5 mL of Folin–Ciocalteau working reagent and 4 mL 7.5% sodium carbonate. Absorbance was measured after one hour at 765 nm. Total phenolic compounds were expressed as gallic acid equivalents (GAE) per gram of raw material and calculated according to the following formula:

GAE = c × V/m, mg/g

where c—gallic acid concentration in mg/mL from the calibration curve; V—the volume in mL; m—the exact weight of the dry material, g.

### 4.5. Determination of Total Proanthocyanidins Content

The total content of proanthocyanidins in the plant raw material was determined using DMCA (p-dimethylaminocinnamaldehyde) reagent [46]. Briefly, 40 μL of each extract was mixed with 4 mL of 0.1% DMCA solution in ethanol acidified with hydrochloric acid. After 15 min, absorbance was measured at 640 nm. Total proanthocyanidins were expressed as epicatechin equivalents (EE) per gram of raw material and calculated according to the following formula:

EE =c × V/m, mg/g

where EE—concentration of total proanthocyanidins in epicatechin equivalents, mg/g; c—epicatechin concentration in mg/mL from the calibration curve; V—the volume in mL; m—the exact weight of the dry material, g.

### 4.6. Determination of Antioxidant Activity

The antioxidant activity of the heather herb extracts was analyzed by spectrophotometric ABTS and FRAP assays. The ABTS assay evaluates the radical scavenging activity and was firstly described by Re et al. [47]. The ABTS assay was performed with modifications described by Raudone et al. [48]. The test solution was prepared by mixing 3 mL of ABTS working solution with 20 μL of each test extract. The mixture was stored in the dark at room temperature for 1 h. The change in absorbance of the mixture was measured with a spectrophotometer at 734 nm. The radical scavenging activity was expressed as antioxidant Trolox equivalents (TE) per gram of raw material and is calculated by the formula:

TE_ABTS_ = c × V/m, mg/g

where c—Trolox concentration in mg/mL from the calibration curve; V—the volume in mL; m—the exact weight of the dry material, g.

The FRAP assay, established by Benzie and Strain (1996) [49] was used to determine the reducing activity in the plant raw material with modifications reported by Raudone et al. [50]. The FRAP working reagent was prepared by mixing reagents of acetate buffer (0.3 M, pH 3.6), TPTZ (0.01 M dissolved in 0.04 M HCl), and FeCl_3_ × 6H_2_O (0.02 M in water) at the ratio of 10:1:1. The test solution was prepared by mixing 3 mL of FRAP working reagent with 20 μL of each test extract. After 1 h, the absorbance of the mixture was measured at 593 nm. The reducing activity was expressed as antioxidant Trolox equivalents (TE) per gram of raw material and was calculated according to the formula:

TE_FRAP_ = c × V/m, mg/g

where c—Trolox concentration in mg/mL from the calibration curve; V—the volume in mL; m—the exact weight of the dry material, g.

### 4.7. Qualitative and Quantitative Analysis by HPLC Method

HPLC analysis was performed using a “Waters e2695 Alliance system” chromatograph with a “Waters 2998” photodiode array detector according to the HPLC methods reported by Raudone et al. [51]. Phenolic compounds were analyzed using ACE Super C18 column (250 mm × 4.6 mm, particle size 3 µm; ACT, UK) with the following mode: A-0.1% trifluoroacetic acid in water and B-acetonitrile, 0 min, 15% B; 0–30 min, 30% B; 30–50 min, 60% B; 50–56 min, 90% B; 56–65 min, 15% B; the flow rate was 0.5 mL/min, injection volume −10 µL, and column temperature −15 °C. Triterpenic compounds, namely ursolic acid, oleanolic acid, corosolic acid, betulinic acid, maslinic acid, uvaol, erythrodiol, and betulin were analyzed using ACE C18 (150 × 4.6 mm, 3 µm) column (ACT, Aberdeen, UK) column with the following isocratic mode of acetonitrile and water (89:11, *v*/*v*), 0.7 mL/min at 20 °C. Triterpenic compounds, namely β-sitosterol, amyrins, friedelin, and lupeol were analyzed using CE C18 (150 × 4.6 mm, 3 µm) column (ACT, Aberdeen, UK) column with the following isocratic mode of acetonitrile and methanol (10:90, *v*/*v*), 1 mL/min at 35 °C. Chromatographic peaks of phenolic and triterpenic compounds were identified by the retention time of the reference compound and the analyte and the UV absorption spectra.

### 4.8. Statistical Analysis

Statistical analysis was performed using Microsoft Office Excel 2010 (Microsoft, Redmond, WA, USA) and SPSS Statistics 27 (IBM, Armonk, NY, USA). During the study, five independent samples were collected from each study habitat and the experiments were repeated three times. Study results were expressed as mean ± standard deviation (SD). Correlations were tested by using the Spearman correlation test. One-way analysis of variance (ANOVA) using the Tukey post hoc criterion was used to assess the statistical significance of the data obtained. Principal component analysis (PCA) was used. The Kaiser–Meyer–Olkin measure of sampling adequacy (KMO > 0.608) and Bartlett’s Test were used to test the suitability of the model (*p* < 0.001). PCA factors with eigenvalues greater than 1 were used. The difference was considered statistically significant at *p* < 0.05. 

## 5. Conclusions

Although variability in the content of bioactive compounds was observed, all samples had low interpopulational variability and similar phytochemical profiles, thus suggesting characteristic phytochemical markers for the heather raw material from the central Europe climatic zone Dfb. The predominant compounds in the plant raw material of heather were chlorogenic acid hyperoside, ursolic, oleanolic acids, and uvaol. The rich phytochemical composition suggests *Calluna vulgaris* aboveground parts as a source of antioxidant active added-value ingredients. 

## Figures and Tables

**Table 1:** Diversification of phenolic compounds in different heather habitats (µg/g).

Compounds	Jankai Forest	Zapyskis Forest	Kalnenai Forest	Sudargas Forest	Gerdziai Forest	Eiciai Forest	Bingeliai Forest	Prienai Forest	Jurasiskes Forest	Paryzines Forest
**4-O-caffeoylquinic acid**	176.07 ± 11.44 a ^1^	226.12 ± 24.72 a,b	231.14 ± 17.22 a,b	242.5 ± 10.97 a,b	233.44 ± 6.79 a,b	237.26 ± 10.85 a,b	267.0 ± 14.23 b	273.76 ± 17.89 b	253.44 ± 9.96 b	264.54 ± 18.23 b
**3,5-dicaffeoylquinic acid**	374.3 ± 58.86 a	372.4 ± 86.46 a	326.8 ± 58.18 a	421.94 ± 78.84 b	248.32 ± 42.80 a	277.92 ± 58.07 a	457.26 ± 102.50 b	492.2 ± 15.69 b	323.66 ± 28.01 a	345.56 ± 71.21 a
**Astragalin**	806.18 ± 129.36 a,b	525.64 ± 95.29 a	1208.9 ± 232.68 b	471.68 ± 163.38 a	342.14 ± 90.64 a	733.64 ± 135.61 a,b	756.36 ± 109.74 a,b	819.58 ± 192.98 a,b	671.80 ± 52.78 a,b	555.6 ± 35.5 a
**Avicularin**	846.2 ± 123.36 a	1214.34 ± 126.55 a,b	1094.86 ± 115.78 a,b	1072.26 ± 164.56 a,b	1054.68 ± 222.05 a,b	1534.96 ± 116.88 a,b	1282.44 ± 198.31 a,b	1183.44 ± 204.25 a,b	1255.28 ± 208.72 a,	1728.04 ± 290.07 b
**Caffeic acid**	45.52 ± 4.29 a	43.28 ± 3.66 a	45.74 ± 4.81 a	47.46 ± 5.93 a	50.42 ± 6.95 a	46.58 ± 3.02 a	48.28 ± 12.35 a	44.34 ± 4.13 a	49.88 ± 2.61 a	47.28 ± 2.41 a
**Chlorogenic acid**	9554.68 ± 586.39 ab	7066.98 ± 1424.99 a	9786.34 ± 1535.42 a	10,091.6 ± 816.44 a	7175.18 ± 481.35 a	8480.86 ± 942.34 a	8173.54 ± 1241.2 a	9074.6 ± 1321.07 a	10,238.18 ± 1043.83 a	11,336.9 ± 1524.90 c
**Epicatechin**	1464.82 ± 261.15 a	625.78 ± 122.76 a	1053.94 ± 299.08 a	1416.68 ± 482.16 a	957.96 ± 359.28 a	637.4 ± 249.56 a,b	1937.14 ± 461.25 a	779.74 ± 238.67 a	1110.24 ± 302.43 a	514.12 ± 196.24 a,b
**Hyperoside**	3609.42 ± 208.61 a	3033.32 ± 374.82 a	2529.54 ± 190.97 a	2663.34 ± 544.71 a	3507.5 ± 146.22 a	3912.32 ± 141.09 a	3186.9 ± 382.63 a	3440.94 ± 634.53 a	3729.24 ± 571.08 a	3951.76 ± 340.93 a
**Isoquercitrin**	1297.66 ± 72.26 a,b	963.34 ± 94.09 a,b	1369.8 ± 138.82 a,b	814.64 ± 209.25 a	1072.92 ± 142.29 a,b	1400.48 ± 112.95 a,b	1250.4 ± 127.19 a,b	1706.94 ± 248.51 b	1451.12 ± 278.59 a,b	1118.88 ± 131.54 a,b
**Kaempferol**	14.74 ± 0.86 a	24.68 ± 4.48 a	35.04 ± 7.52 a	121.26 ± 15.50 b	21.38 ± 8.92 a	109.46 ± 21.33 b	33.76 ± 3.62 a	30.96 ± 5.63 a	26.14 ± 1.9 a	30.26 ± 5.36 a
**Neochlorogenic acid**	2247.9 ± 336.44 a	1805.94 ± 230.09 a	2573.32 ± 308.81 a	2372.76 ± 126.12 a	2082.6 ± 305.16 a	2281.88 ± 247.32 a	2620.82 ± 286.24 a	2589.66± 437.94 a	2191.48 ± 254.42 a	2256.62 ± 440.37 a
**Proanthocyanidin A1**	324.42 ± 23.48 a	284.48 ± 13.98 a	327.28 ± 32.46 a	408.06 ± 77.94 a	305.6 ± 46.62 a	253.14 ± 34.29 a	248.44 ± 46.72 a	408.92 ± 82.54 a	357.38 ± 55.37 a	247.68 ± 24.55 a
**Proanthocyanidin B2**	1245.48 ± 102.53 a,b	801.28 ± 81.31 a	1121.42 ± 178.18 a	1341.78 ± 124.06 b	831.34 ± 74.83 a	1029.22 ± 119.24 a,b	1339.4 ± 277.51 b	877.04 ± 87.27 a	1258.4 ± 295.14 a,b	944.16 ± 123.59 a
**Proanthocyanidin B3**	213.66 ± 3.57 a,b	168.04 ± 23.03 a,b	157.96 ± 29.06 a,b	214.12 ± 17.05 a,b	206.1 ± 3.95 a,b	272.42 ± 65.51 b	131.06 ± 19.75 a	133.4 ± 15.49 a	164.66 ± 20.85 a,b	211.86 ± 27.07 a,b
**Protocatechuic acid**	35.15 ± 1.74 a	36.5 ± 4.41 a,b	38.3 ± 3.56 a,b	65.84 ± 11.13 b	38.52 ± 3.45 a,b	61.5 ± 11.45 a,b	34.74 ± 3.56 a	35.96 ± 2.79 a	36.16 ± 3.99 a	40.32 ± 7.36 a,b
**Quercetin**	507.52 ± 43.86 a	429.02 ± 35.12 a	543.38 ± 88.63 a	1338.56 ± 147.59 b	601.04 ± 88.85 a	1362.78 ± 145.48 b	331.52 ± 38.81 a	417.92 ± 28.71 a	412.22 ± 32.06 a	514.04 ± 54.34 a
**Quercetin-3-arabinopyranoside**	511.46 ± 63.69 a	646.06 ± 84.58 a,b	614.44 ± 87.66 a,b	583.22 ± 100.86 a,b	664.22 ± 98.38 a,b	906.98 ± 95.67 a,b	736.7 ± 110.25 a,b	623.2 ± 115.31 a,b	821.7 ± 137.63 a,b	1028.18 ± 163.54 b

Different letters indicate statistically significant differences in the content of phenolic compounds between different heather habitats.

**Table 2:** Diversification of triterpenic compounds in different heather habitats (µg/g).

Compounds	Jankai Forest	Zapyskis Forest	Kalnenai Forest	Sudargas Forest	Gerdziai Forest	Eiciai Forest	Bingeliai Forest	Prienai Forest	Jurasiskes Forest	Paryzines Forest
**Oleanolic acid**	3794.29 ± 329.25 a	4355.32 ± 184.21 a	3527.23 ± 293.23 a	4193.68 ± 242.22 a	4795.64 ± 146.05 a	4195.19 ± 300.38 a	4570.66 ± 98.43 a	4441.53 ± 375.90 a	3818.73 ± 322.03 a	3982.31 ± 428.49 a
**Ursolic acid**	9700.96 ± 725.95 a,b	9981.77 ± 292.59 a,b	8181.79 ± 716.58 a	10,379.29 ± 403.74 a,b	9785.82 ± 580.90 a,b	10,406.46 ± 460.76 b	10,709.56 ± 391.94 b	10,274.12 ± 273.65 b	8915.74 ± 235.33 a	9974.18 ± 296.99 a,b
**Maslinic acid**	355.18 ± 46.79 a	450.47 ± 43.82 a	425.74 ± 70.48 a	488.72 ± 90.69 a	422.44 ± 80.12 a	454,91 ± 56,70 a	733.07 ± 100.81 a	707.53 ± 153.45 a	481.36 ± 103.75 a	389.82 ± 69.30 a
**Corosolic acid**	411.32 ± 50.61 a	793.66 ± 191.37 a	392.51 ± 53.59 a	399.43 ± 38.82 a	337.46 ± 79.38 a	398.77 ± 52.42 a	742.62 ± 168.13 a	698.48 ± 165.27 a	455.93 ± 82.62 a	398.08 ± 73.66 a
**Betulinic acid**	733.27 ± 85.81 a	812.32 ± 149.08 a	790.74 ± 143.59 a	673.51 ± 84.45 a	404.34 ± 86.42 b	588.68 ± 83.97 a,b	495.39 ± 66.38 b	582.77 ± 176.33 a,b	603.93 ± 88.12 a	405.71 ± 49.54 b
**Betulin**	355.15 ± 27.87 a,b,c	462.04 ± 22.43 b,c	518.46 ± 94.48 b,c	422.98 ± 20.09 a,b,c	551.69 ± 58.57 c	433.46 ± 53.98 a,b,c	244.71 ± 16.86 a	361.80 ± 31.46 a,b,c	316.62 ± 26.47 a,b	381.36 ± 31.02 a,b,c
**Erythrodiol**	233.86 ± 21.72 a,b	323.25 ± 47.58 a,b,c	164.56 ± 14.84 a	397.07 ± 42.66 c	310.99 ± 24.56 a,b,c	255.74 ± 15.69 a,b,c	225.18 ± 13.05 a,b	249.41 ± 20.04 a,b,c	255.49 ± 48.92 a,b,c	329.15 ± 55.61 b,c
**Uvaol**	1223.99 ± 207.77 a,b	2450.64 ± 159.76 c	917.87 ± 120.98 a	1773.11 ± 159.82 b,c	1187.79 ± 71.41 a,b	1136.68 ± 113.51 a,b	1570.03 ± 66.36 a,b	1888.68 ± 339.91 b,c	1254.48 ± 115.59 a,b	1223.68 ± 203.67 a,b
**Lupeol**	0.27 ± 0.27 a	7.45 ± 4.36 a	2.77 ± 2.01 a	16.90 ± 6.82 a	14.08 ± 3.54 a	7.49 ± 1.38 a	3.01 ± 2.01 a	15.73 ± 2.63 a	12.67 ± 9.29 a	3.98 ± 2.59 a
**β-Amyrin**	114.52 ± 8.71 a	134.87 ± 14.59 a	113.02 ± 13.43 a	121.91 ± 14.31 a	159.63 ± 23.19 a	123.33 ± 13.75 a	173.37 ± 24.82 a	107.93 ± 16.76 a	144.83 ± 8.6 a	129.32 ± 23.16 a
**β-Sitosterol**	382.73 ± 8.89 b	321.65 ± 24.95 a,b	315.13 ± 30.72 a,b	372.94 ± 18.54 b	279.63 ± 34.07 a,b	304.59 ± 20.81 a,b	251.83 ± 20.67 a	344.09 ± 20.08 a,b	300.93 ± 19.41 a,b	334.61 ± 18.96 a,b
**α-Amyrin**	475.05 ± 82.21 a,b,c	912.15 ± 89.02 b,c	224.87 ± 35.26 a	731.46 ± 131.65 a,b,c	545.07 ± 66.66 a,b,c	415.99 ± 92.09 a,b	375.68 ± 50.22 a,b	1023.75 ± 282.95 c	724.66 ± 86.65 a,b,c	637.12 ± 64.63 a,b,c
**Friedelin**	98.10 ± 23.68 a	97.49 ± 13.36 a	85.52 ± 20.86 a	178.02 ± 20.08 a,b	221.33 ± 45.37 b	190.17 ± 24.22 a,b	92.15 ± 18.84 a	217.31 ± 23.59 b	90.34 ± 11.08 a	127.42 ± 30.28 a,b

Different letters indicate statistically significant differences in the content of triterpenic compounds between different heather habitats.

**Table 3:** Plant raw material habitats of heather.

Population Number	Heather Growth Site (Forest Name and Region)	Plant Raw Material Habitat	WGS Coordinates	Altitude
1	Jankai Forest (Kazlu Ruda municipality)	Forest	54°49′, 23°22′	66.0 m
2	Zapyskis Forest (Kaunas district)	Outskirts	54°54′, 23°38′	66.9 m
3	Kalnenai Forest (Jurbarkas district)	Outskirts	55°04′, 22°41′	34.0 m
4	Sudargas Forest (Sakiai district)	Forest	55°01′, 22°39′	42.1 m
5	Gerdziai Forest (Sakiai district)	Outskirts	54°57′, 23°23′	70.9 m
6	Eiciai Forest (Taurages district)	Forest	55°08′, 22°31′	42.7 m
7	Bingeliai Forest (Varenos district)	Forest	54°09′, 24°16′	111.8 m
8	Prienai Forest (Prienai district)	Outskirts	54°34′, 23°55′	106.4 m
9	Jurasiskes Forest (Druskininkai municipality)	Forest	54°06′, 23°55′	135.3 m
10	Paryzines Forest (Sakiai district)	Outskirts	54°57′, 23°22′	72.8 m

## Data Availability

All data generated during this study are included in this article.

## Supplementary Materials

The following supporting information can be downloaded at: https://www.mdpi.com/article/10.3390/plants11172207/s1, Table S1. Average air temperatures (°C) during the vegetation period (April–September 2020) in the areas of investigated populations of C. vulgaris; Table S2. The amounts of precipitation (mm) during the vegetation period (April–September 2020) in the areas of investigated populations of C. vulgaris; Table S3. The sunshine duration (h) during the vegetation period (April–September 2020) in the areas of investigated populations of C. vulgaris; Table S4. The soil type and soil pH in the areas of investigated populations of *C. vulgaris*.
