# Supplementary material for: Phytochemical Diversity and Antioxidant Potential of Wild Heather (Calluna vulgaris L.) Aboveground Parts
Source: Plants (Basel). 2022 Aug 25;11(17):2207. doi: 10.3390/plants11172207 (PMC9460699; doi:10.3390/plants11172207)
Supplement: Supplementary file 1 [file plants-11-02207-s001.zip › plants-1825385-supplementary.pdf]

Table S1. Average air temperatures (°C) during the vegetation period (April–September, 2020) in the areas of investigated populations of *C. vulgaris*.

Population number	Heather growth site (forest name and region)	April	May	June	July	August
1	Jankai forest (Kazlu Ruda municipality)	6.7	10.7	18.7	17.7	19.3
2	Zapyskis forest (Kaunas district)	5.7	10.7	19.3	17.3	19.3
3	Kalnenai forest (Jurbarkas district)	5.7	10.7	19.3	17.3	19.3
4	Sudargas forest (Sakiai district)	5.7	10.7	19.3	17.3	19.3
5	Gerdziai forest (Sakiai district)	5.7	10.7	19.3	17.3	19.3
6	Eiciai forest (Taurages district)	5.7	10.7	19.3	17.3	19.3
7	Bingeliai forest (Varenos district)	6.7	10.3	19.3	17.7	19.3
8	Prienai forest (Prienai district)	6.7	10.7	19.3	17.7	19.3
9	Jurasiskes forest (Druskininkai municipality)	5.3	10.3	19.3	17.7	18.7
10	Paryzines forest (Sakiai district)	5.7	10.7	19.3	17.3	19.3

Table S2. The amounts of precipitation (mm) during the vegetation period (April–September, 2020) in the areas of investigated populations of *C. vulgaris*.

Population number	Heather growth site (forest name and region)	April	May	June	July	August
1	Jankai forest (Kazlu Ruda municipality)	30 - 45	60 - 90	75 - 100	30 - 60	80 - 100
2	Zapyskis forest (Kaunas district)	30 - 45	90 - 120	75 - 100	30 - 60	80 - 100
3	Kalnenai forest (Jurbarkas district)	30 - 45	60 - 90	75 - 100	60 - 90	40 - 60
4	Sudargas forest (Sakiai district)	30 - 45	60 - 90	75 - 100	30 - 60	60 - 80
5	Gerdziai forest (Sakiai district)	30 - 45	60 - 90	75 - 100	30 - 60	60 - 80
6	Eiciai forest (Taurages district)	30 - 45	30 - 60	100 - 125	60 - 90	40 - 60
7	Bingeliai forest (Varenos district)	30 - 45	60 - 90	150 - 175	10 - 30	100 - 127
8	Prienai forest (Prienai district)	30 - 45	90 - 120	175 - 202	10 - 30	100 - 127
9	Jurasiskes forest (Druskininkai municipality)	30 - 45	60 - 90	75 - 100	10 - 30	80 - 100
10	Paryzines forest (Sakiai district)	30 - 45	60 - 90	75 - 100	30 - 60	60 - 80

Table S3. The sunshine duration (h) during the vegetation period (April–September, 2020) in the areas of investigated populations of *C. vulgaris*.

Population number	Heather growth site (forest name and region)	April	May	June	July	August
1	Jankai forest (Kazlu Ruda municipality)	230 - 250	250 - 275	260 - 270	250 - 270	270 - 279
2	Zapyskis forest (Kaunas district)	230 - 250	250 - 275	260 - 270	250 - 270	270 - 279
3	Kalnenai forest (Jurbarkas district)	230 - 250	250 - 275	260 - 270	250 - 270	260 - 270
4	Sudargas forest (Sakiai district)	230 - 250	250 - 275	260 - 270	250 - 270	260 - 270
5	Gerdziai forest (Sakiai district)	230 - 250	250 - 275	260 - 270	250 - 270	260 - 270
6	Eiciai forest (Taurages district)	230 - 250	275 - 300	260 - 270	250 - 270	260 - 270

7	Bingeliai forest (Varenos district)	169 - 190	250 - 275	260 - 270	250 - 270	250 - 260
8	Prienai forest (Prienai district)	190 - 210	250 - 275	260 - 270	250 - 270	260 - 270
9	Jurasiskes forest (Druskininkai municipality)	169 - 190	250 - 275	260 - 270	230 - 250	250 - 260
10	Paryzines forest (Sakiai district)	230 - 250	250 - 275	260 - 270	250 - 270	260 - 270

Table S4. The soil type and soil pH in the areas of investigated populations of *C. vulgaris*.

Population number	Heather growth site (forest name and region)	soil pH	soil type
1	Jankai forest (Kazlu Ruda municipality)	6.7 - 7.0	Glacial till
2	Zapyskis forest (Kaunas district)	6.7 - 7.0	Glaciolacustrine clay and loam
3	Kalnenai forest (Jurbarkas district)	6.7 - 7.0	Glaciolacustrine clay and loam
4	Sudargas forest (Sakiai district)	6.7 - 7.0	Glaciofluvial sand
5	Gerdziai forest (Sakiai district)	6.7 - 7.0	Glaciofluvial sand
6	Eiciai forest (Taurages district)	6.1 - 5.5	Glaciofluvial sand
7	Bingeliai forest (Varenos district)	< 4.5	Glaciofluvial sand
8	Prienai forest (Prienai district)	5.6 - 6.0	Glaciolacustrine clay and loam
9	Jurasiskes forest (Druskininkai municipality)	< 4.5	Glaciofluvial sand
10	Paryzines forest (Sakiai district)	6.7 - 7.0	Glaciofluvial sand
